# Supplementary material for: Transcriptome Analysis Reveals Vimentin-Induced Disruption of Cell–Cell Associations Augments Breast Cancer Cell Migration
Source: Cells. 2022 Dec 13;11(24):4035. doi: 10.3390/cells11244035 (PMC9776984; doi:10.3390/cells11244035)
Supplement: Supplementary file 1 [file cells-11-04035-s001.zip › DEGs and enrichment analysis.zip!/DEGs and enrichment analysis/WT_VIMvsVector KEGG/DOWN/WT_VIMvsVector_DOWN.pathway_map.zip!/WT_VIMvsVector_DOWN.pathway_map/WT_VIMvsVector_down_KEGGenrich.xls_rendered_html_detail.html]

Pathway Enrichment


  

# The most enriched pathway terms

Statistic method: hypergeometric test

FDR correction method: Benjamini and Hochberg

| Term | Description | Sample number | Background number | P-value | Corrected P-value | Gene\_id | Gene\_name | KEGG\_ID |
| --- | --- | --- | --- | --- | --- | --- | --- | --- |
| hsa05146 | Amoebiasis | 9\|118 | 94\|9228 | 2.87759099785324e-06 | 0.000589906154559914 | ENSG00000169429 ENSG00000058085 ENSG00000092969 ENSG00000188153 ENSG00000169031 ENSG00000081041 ENSG00000135862 ENSG00000053747 ENSG00000137462 | CXCL8 LAMC2 TGFB2 COL4A5 COL4A3 CXCL2 LAMC1 LAMA3 TLR2 | hsa:3576 hsa:3918 hsa:7042 hsa:1287 hsa:1285 hsa:2920 hsa:3915 hsa:3909 hsa:7097 |
| hsa00830 | Retinol metabolism | 5\|118 | 57\|9228 | 0.000773551440281187 | 0.0672460105310965 | ENSG00000162496 ENSG00000128918 ENSG00000003137 ENSG00000256612 ENSG00000095596 | DHRS3 ALDH1A2 CYP26B1 CYP2B7P CYP26A1 | hsa:9249 hsa:8854 hsa:56603 hsa:1555 hsa:1592 |
| hsa04512 | ECM-receptor interaction | 6\|118 | 91\|9228 | 0.00105947116815198 | 0.0672460105310965 | ENSG00000058085 ENSG00000188153 ENSG00000169031 ENSG00000135862 ENSG00000053747 ENSG00000115221 | LAMC2 COL4A5 COL4A3 LAMC1 LAMA3 ITGB6 | hsa:3918 hsa:1287 hsa:1285 hsa:3915 hsa:3909 hsa:3694 |
| hsa00982 | Drug metabolism - cytochrome P450 | 5\|118 | 64\|9228 | 0.00131211727865554 | 0.0672460105310965 | ENSG00000213366 ENSG00000259583 ENSG00000184254 ENSG00000256612 ENSG00000241720 | GSTM2 AC015712.2 ALDH1A3 CYP2B7P AL158847.1 | hsa:2946 hsa:220 hsa:220 hsa:1555 hsa:2947 |
| hsa00980 | Metabolism of xenobiotics by cytochrome P450 | 5\|118 | 69\|9228 | 0.00183862068740863 | 0.0753834481837536 | ENSG00000213366 ENSG00000259583 ENSG00000184254 ENSG00000256612 ENSG00000241720 | GSTM2 AC015712.2 ALDH1A3 CYP2B7P AL158847.1 | hsa:2946 hsa:220 hsa:220 hsa:1555 hsa:2947 |
| hsa04621 | NOD-like receptor signaling pathway | 8\|118 | 195\|9228 | 0.00347097458541138 | 0.116669769505841 | ENSG00000169429 ENSG00000117226 ENSG00000162645 ENSG00000139112 ENSG00000111335 ENSG00000081041 ENSG00000023445 ENSG00000132357 | CXCL8 GBP3 GBP2 GABARAPL1 OAS2 CXCL2 BIRC3 CARD6 | hsa:3576 hsa:2635 hsa:2634 hsa:23710 hsa:4939 hsa:2920 hsa:330 hsa:84674 |
| hsa05222 | Small cell lung cancer | 6\|118 | 121\|9228 | 0.00448426601108739 | 0.116669769505841 | ENSG00000058085 ENSG00000188153 ENSG00000169031 ENSG00000135862 ENSG00000053747 ENSG00000023445 | LAMC2 COL4A5 COL4A3 LAMC1 LAMA3 BIRC3 | hsa:3918 hsa:1287 hsa:1285 hsa:3915 hsa:3909 hsa:330 |
| hsa04510 | Focal adhesion | 9\|118 | 249\|9228 | 0.00455296661486208 | 0.116669769505841 | ENSG00000058085 ENSG00000128052 ENSG00000162909 ENSG00000188153 ENSG00000169031 ENSG00000135862 ENSG00000053747 ENSG00000023445 ENSG00000115221 | LAMC2 KDR CAPN2 COL4A5 COL4A3 LAMC1 LAMA3 BIRC3 ITGB6 | hsa:3918 hsa:3791 hsa:824 hsa:1287 hsa:1285 hsa:3915 hsa:3909 hsa:330 hsa:3694 |
| hsa05145 | Toxoplasmosis | 6\|118 | 125\|9228 | 0.00525256357012163 | 0.119641725763882 | ENSG00000058085 ENSG00000092969 ENSG00000135862 ENSG00000053747 ENSG00000023445 ENSG00000137462 | LAMC2 TGFB2 LAMC1 LAMA3 BIRC3 TLR2 | hsa:3918 hsa:7042 hsa:3915 hsa:3909 hsa:330 hsa:7097 |
| hsa05144 | Malaria | 3\|118 | 33\|9228 | 0.00841056591507688 | 0.172416601259076 | ENSG00000169429 ENSG00000092969 ENSG00000137462 | CXCL8 TGFB2 TLR2 | hsa:3576 hsa:7042 hsa:7097 |
| hsa04933 | AGE-RAGE signaling pathway in diabetic complications | 5\|118 | 102\|9228 | 0.00977378455804267 | 0.182147803127159 | ENSG00000078401 ENSG00000169429 ENSG00000092969 ENSG00000188153 ENSG00000169031 | EDN1 CXCL8 TGFB2 COL4A5 COL4A3 | hsa:1906 hsa:3576 hsa:7042 hsa:1287 hsa:1285 |
| hsa05204 | Chemical carcinogenesis | 4\|118 | 72\|9228 | 0.0134096306909963 | 0.229081190971187 | ENSG00000213366 ENSG00000259583 ENSG00000184254 ENSG00000241720 | GSTM2 AC015712.2 ALDH1A3 AL158847.1 | hsa:2946 hsa:220 hsa:220 hsa:2947 |
| hsa05323 | Rheumatoid arthritis | 4\|118 | 75\|9228 | 0.0153888962802559 | 0.242671056727112 | ENSG00000169429 ENSG00000092969 ENSG00000081041 ENSG00000137462 | CXCL8 TGFB2 CXCL2 TLR2 | hsa:3576 hsa:7042 hsa:2920 hsa:7097 |
| hsa05321 | Inflammatory bowel disease (IBD) | 3\|118 | 44\|9228 | 0.0184498930317636 | 0.27015914796511 | ENSG00000092969 ENSG00000138378 ENSG00000137462 | TGFB2 STAT4 TLR2 | hsa:7042 hsa:6775 hsa:7097 |
| hsa04151 | PI3K-Akt signaling pathway | 10\|118 | 376\|9228 | 0.0219791736816922 | 0.273983056328409 | ENSG00000157613 ENSG00000058085 ENSG00000128052 ENSG00000109321 ENSG00000188153 ENSG00000169031 ENSG00000135862 ENSG00000053747 ENSG00000115221 ENSG00000137462 | CREB3L1 LAMC2 KDR AREG COL4A5 COL4A3 LAMC1 LAMA3 ITGB6 TLR2 | hsa:90993 hsa:3918 hsa:3791 hsa:374 hsa:1287 hsa:1285 hsa:3915 hsa:3909 hsa:3694 hsa:7097 |
| hsa04080 | Neuroactive ligand-receptor interaction | 7\|118 | 219\|9228 | 0.022078897568748 | 0.273983056328409 | ENSG00000078401 ENSG00000135346 ENSG00000136928 ENSG00000148926 ENSG00000168398 ENSG00000065325 ENSG00000164418 | EDN1 CGA GABBR2 ADM BDKRB2 GLP2R GRIK2 | hsa:1906 hsa:1081 hsa:9568 hsa:133 hsa:624 hsa:9340 hsa:2898 |
| hsa04514 | Cell adhesion molecules (CAMs) | 5\|118 | 128\|9228 | 0.0239464941588587 | 0.273983056328409 | ENSG00000179776 ENSG00000173482 ENSG00000134258 ENSG00000107738 ENSG00000188404 | CDH5 PTPRM VTCN1 VSIR SELL | hsa:1003 hsa:5797 hsa:79679 hsa:64115 hsa:6402 |
| hsa00360 | Phenylalanine metabolism | 2\|118 | 19\|9228 | 0.0240570488483481 | 0.273983056328409 | ENSG00000259583 ENSG00000184254 | AC015712.2 ALDH1A3 | hsa:220 hsa:220 |
| hsa04926 | Relaxin signaling pathway | 5\|118 | 133\|9228 | 0.0276806026625066 | 0.298659133990203 | ENSG00000078401 ENSG00000157613 ENSG00000188153 ENSG00000169031 ENSG00000137745 | EDN1 CREB3L1 COL4A5 COL4A3 MMP13 | hsa:1906 hsa:90993 hsa:1287 hsa:1285 hsa:4322 |
| hsa05142 | Chagas disease (American trypanosomiasis) | 4\|118 | 93\|9228 | 0.0310765036923797 | 0.318534162846892 | ENSG00000169429 ENSG00000092969 ENSG00000168398 ENSG00000137462 | CXCL8 TGFB2 BDKRB2 TLR2 | hsa:3576 hsa:7042 hsa:624 hsa:7097 |
| hsa05418 | Fluid shear stress and atherosclerosis | 6\|118 | 188\|9228 | 0.0333217658244088 | 0.325283904476372 | ENSG00000179776 ENSG00000078401 ENSG00000213366 ENSG00000128052 ENSG00000116701 ENSG00000241720 | CDH5 EDN1 GSTM2 KDR NCF2 AL158847.1 | hsa:1003 hsa:1906 hsa:2946 hsa:3791 hsa:4688 hsa:2947 |
| hsa04270 | Vascular smooth muscle contraction | 5\|118 | 146\|9228 | 0.039057728048239 | 0.358003463442365 | ENSG00000078401 ENSG00000185532 ENSG00000122786 ENSG00000148926 ENSG00000188257 | EDN1 PRKG1 CALD1 ADM PLA2G2A | hsa:1906 hsa:5592 hsa:800 hsa:133 hsa:5320 |
| hsa00340 | Histidine metabolism | 2\|118 | 25\|9228 | 0.0401662422398751 | 0.358003463442365 | ENSG00000259583 ENSG00000184254 | AC015712.2 ALDH1A3 | hsa:220 hsa:220 |
| hsa04061 | Viral protein interaction with cytokine and cytokine receptor | 3\|118 | 61\|9228 | 0.0429326596207711 | 0.366716467594086 | ENSG00000169429 ENSG00000006606 ENSG00000081041 | CXCL8 CCL26 CXCL2 | hsa:3576 hsa:10344 hsa:2920 |
| hsa05202 | Transcriptional misregulation in cancer | 6\|118 | 205\|9228 | 0.0474949280803364 | 0.389458410258759 | ENSG00000164120 ENSG00000169429 ENSG00000146674 ENSG00000023445 ENSG00000058091 ENSG00000132170 | HPGD CXCL8 IGFBP3 BIRC3 CDK14 PPARG | hsa:3248 hsa:3576 hsa:3486 hsa:330 hsa:5218 hsa:5468 |
| hsa04024 | cAMP signaling pathway | 6\|118 | 214\|9228 | 0.0563137760944766 | 0.441399961375488 | ENSG00000125257 ENSG00000078401 ENSG00000157613 ENSG00000135346 ENSG00000136928 ENSG00000125398 | ABCC4 EDN1 CREB3L1 CGA GABBR2 SOX9 | hsa:10257 hsa:1906 hsa:90993 hsa:1081 hsa:9568 hsa:6662 |
| hsa04216 | Ferroptosis | 3\|118 | 69\|9228 | 0.0581356046689668 | 0.441399961375488 | ENSG00000047457 ENSG00000130066 ENSG00000240216 | CP SAT1 CPHL1P | hsa:1356 hsa:6303 hsa:1356 |
| hsa04610 | Complement and coagulation cascades | 3\|118 | 71\|9228 | 0.0622844869239691 | 0.456011422121917 | ENSG00000237248 ENSG00000175899 ENSG00000168398 | LINC00987 A2M BDKRB2 | hsa:2 hsa:2 hsa:624 |
| hsa00410 | beta-Alanine metabolism | 2\|118 | 34\|9228 | 0.069774558540837 | 0.493233948305917 | ENSG00000259583 ENSG00000184254 | AC015712.2 ALDH1A3 | hsa:220 hsa:220 |
| hsa04668 | TNF signaling pathway | 4\|118 | 126\|9228 | 0.0777790561894633 | 0.513757032010961 | ENSG00000078401 ENSG00000157613 ENSG00000081041 ENSG00000023445 | EDN1 CREB3L1 CXCL2 BIRC3 | hsa:1906 hsa:90993 hsa:2920 hsa:330 |
| hsa05161 | Hepatitis B | 5\|118 | 179\|9228 | 0.0792297920843903 | 0.513757032010961 | ENSG00000157613 ENSG00000169429 ENSG00000092969 ENSG00000138378 ENSG00000137462 | CREB3L1 CXCL8 TGFB2 STAT4 TLR2 | hsa:90993 hsa:3576 hsa:7042 hsa:6775 hsa:7097 |
| hsa05140 | Leishmaniasis | 3\|118 | 79\|9228 | 0.0801962196309793 | 0.513757032010961 | ENSG00000092969 ENSG00000116701 ENSG00000137462 | TGFB2 NCF2 TLR2 | hsa:7042 hsa:4688 hsa:7097 |
| hsa00350 | Tyrosine metabolism | 2\|118 | 39\|9228 | 0.0884921556047782 | 0.549723996938774 | ENSG00000259583 ENSG00000184254 | AC015712.2 ALDH1A3 | hsa:220 hsa:220 |
| hsa01524 | Platinum drug resistance | 3\|118 | 88\|9228 | 0.102686305901866 | 0.611520924337836 | ENSG00000213366 ENSG00000023445 ENSG00000241720 | GSTM2 BIRC3 AL158847.1 | hsa:2946 hsa:330 hsa:2947 |
| hsa00860 | Porphyrin and chlorophyll metabolism | 2\|118 | 43\|9228 | 0.104406011472313 | 0.611520924337836 | ENSG00000047457 ENSG00000240216 | CP CPHL1P | hsa:1356 hsa:1356 |
| hsa04012 | ErbB signaling pathway | 3\|118 | 94\|9228 | 0.118918382873189 | 0.636159479152636 | ENSG00000109321 ENSG00000174808 ENSG00000114423 | AREG BTC CBLB | hsa:374 hsa:685 hsa:868 |
| hsa04657 | IL-17 signaling pathway | 3\|118 | 94\|9228 | 0.118918382873189 | 0.636159479152636 | ENSG00000169429 ENSG00000137745 ENSG00000081041 | CXCL8 MMP13 CXCL2 | hsa:3576 hsa:4322 hsa:2920 |
| hsa04218 | Cellular senescence | 5\|118 | 204\|9228 | 0.120276718044329 | 0.636159479152636 | ENSG00000169429 ENSG00000146674 ENSG00000162909 ENSG00000092969 ENSG00000185650 | CXCL8 IGFBP3 CAPN2 TGFB2 ZFP36L1 | hsa:3576 hsa:3486 hsa:824 hsa:7042 hsa:677 |
| hsa04962 | Vasopressin-regulated water reabsorption | 2\|118 | 47\|9228 | 0.121025461887575 | 0.636159479152636 | ENSG00000157613 ENSG00000111348 | CREB3L1 ARHGDIB | hsa:90993 hsa:397 |
| hsa04060 | Cytokine-cytokine receptor interaction | 5\|118 | 208\|9228 | 0.127632357181457 | 0.651184921109489 | ENSG00000181634 ENSG00000169429 ENSG00000092969 ENSG00000006606 ENSG00000081041 | TNFSF15 CXCL8 TGFB2 CCL26 CXCL2 | hsa:9966 hsa:3576 hsa:7042 hsa:10344 hsa:2920 |
| hsa04064 | NF-kappa B signaling pathway | 3\|118 | 98\|9228 | 0.130236984221898 | 0.651184921109489 | ENSG00000169429 ENSG00000081041 ENSG00000023445 | CXCL8 CXCL2 BIRC3 | hsa:3576 hsa:2920 hsa:330 |
| hsa02010 | ABC transporters | 2\|118 | 51\|9228 | 0.13824107283116 | 0.673728551619015 | ENSG00000125257 ENSG00000198691 | ABCC4 ABCA4 | hsa:10257 hsa:24 |
| hsa00072 | Synthesis and degradation of ketone bodies | 1\|118 | 12\|9228 | 0.143180727326895 | 0.673728551619015 | ENSG00000134240 | HMGCS2 | hsa:3158 |
| hsa04211 | Longevity regulating pathway | 3\|118 | 103\|9228 | 0.144896055197371 | 0.673728551619015 | ENSG00000157613 ENSG00000109819 ENSG00000132170 | CREB3L1 PPARGC1A PPARG | hsa:90993 hsa:10891 hsa:5468 |
| hsa00010 | Glycolysis / Gluconeogenesis | 3\|118 | 104\|9228 | 0.147891633282223 | 0.673728551619015 | ENSG00000259583 ENSG00000184254 ENSG00000186197 | AC015712.2 ALDH1A3 EDARADD | hsa:220 hsa:220 hsa:2023 |
| hsa05410 | Hypertrophic cardiomyopathy (HCM) | 3\|118 | 106\|9228 | 0.153943074691028 | 0.680218150177075 | ENSG00000078401 ENSG00000092969 ENSG00000115221 | EDN1 TGFB2 ITGB6 | hsa:1906 hsa:7042 hsa:3694 |
| hsa04923 | Regulation of lipolysis in adipocytes | 2\|118 | 55\|9228 | 0.155952453943037 | 0.680218150177075 | ENSG00000185532 ENSG00000135346 | PRKG1 CGA | hsa:5592 hsa:1081 |
| hsa04961 | Endocrine and other factor-regulated calcium reabsorption | 2\|118 | 56\|9228 | 0.160446809354442 | 0.68524158161793 | ENSG00000197959 ENSG00000168398 | DNM3 BDKRB2 | hsa:26052 hsa:624 |
| hsa04950 | Maturity onset diabetes of the young | 1\|118 | 14\|9228 | 0.164982556062485 | 0.690233142710395 | ENSG00000164749 | HNF4G | hsa:3174 |
| hsa04380 | Osteoclast differentiation | 3\|118 | 116\|9228 | 0.18529361209239 | 0.743198636791102 | ENSG00000092969 ENSG00000116701 ENSG00000132170 | TGFB2 NCF2 PPARG | hsa:7042 hsa:4688 hsa:5468 |
| hsa00480 | Glutathione metabolism | 2\|118 | 62\|9228 | 0.187868138824179 | 0.743198636791102 | ENSG00000213366 ENSG00000241720 | GSTM2 AL158847.1 | hsa:2946 hsa:2947 |
| hsa05134 | Legionellosis | 3\|118 | 117\|9228 | 0.18851867860067 | 0.743198636791102 | ENSG00000169429 ENSG00000081041 ENSG00000137462 | CXCL8 CXCL2 TLR2 | hsa:3576 hsa:2920 hsa:7097 |
| hsa05165 | Human papillomavirus infection | 7\|118 | 377\|9228 | 0.207477055059741 | 0.790247199772527 | ENSG00000157613 ENSG00000058085 ENSG00000188153 ENSG00000169031 ENSG00000135862 ENSG00000053747 ENSG00000115221 | CREB3L1 LAMC2 COL4A5 COL4A3 LAMC1 LAMA3 ITGB6 | hsa:90993 hsa:3918 hsa:1287 hsa:1285 hsa:3915 hsa:3909 hsa:3694 |
| hsa04922 | Glucagon signaling pathway | 3\|118 | 123\|9228 | 0.208162677013251 | 0.790247199772527 | ENSG00000157613 ENSG00000109819 ENSG00000275993 | CREB3L1 PPARGC1A SIK1B | hsa:90993 hsa:10891 hsa:102724428 |
| hsa04929 | GnRH secretion | 2\|118 | 68\|9228 | 0.215879250142451 | 0.804640841440044 | ENSG00000135346 ENSG00000136928 | CGA GABBR2 | hsa:1081 hsa:9568 |
| hsa00590 | Arachidonic acid metabolism | 2\|118 | 71\|9228 | 0.230030760734064 | 0.810927921279892 | ENSG00000256612 ENSG00000188257 | CYP2B7P PLA2G2A | hsa:1555 hsa:5320 |
| hsa04390 | Hippo signaling pathway | 4\|118 | 193\|9228 | 0.233613301330891 | 0.810927921279892 | ENSG00000118523 ENSG00000092969 ENSG00000109321 ENSG00000023445 | CTGF TGFB2 AREG BIRC3 | hsa:1490 hsa:7042 hsa:374 hsa:330 |
| hsa04977 | Vitamin digestion and absorption | 1\|118 | 21\|9228 | 0.237045905929683 | 0.810927921279892 | ENSG00000086205 | FOLH1 | hsa:2346 |
| hsa04918 | Thyroid hormone synthesis | 2\|118 | 73\|9228 | 0.239500686361345 | 0.810927921279892 | ENSG00000157613 ENSG00000135346 | CREB3L1 CGA | hsa:90993 hsa:1081 |
| hsa04976 | Bile secretion | 2\|118 | 73\|9228 | 0.239500686361345 | 0.810927921279892 | ENSG00000125257 ENSG00000080493 | ABCC4 SLC4A4 | hsa:10257 hsa:8671 |
| hsa05164 | Influenza A | 4\|118 | 196\|9228 | 0.241898041987664 | 0.810927921279892 | ENSG00000137648 ENSG00000169429 ENSG00000111335 ENSG00000134321 | TMPRSS4 CXCL8 OAS2 RSAD2 | hsa:56649 hsa:3576 hsa:4939 hsa:91543 |
| hsa04152 | AMPK signaling pathway | 3\|118 | 134\|9228 | 0.245256249362699 | 0.810927921279892 | ENSG00000157613 ENSG00000109819 ENSG00000132170 | CREB3L1 PPARGC1A PPARG | hsa:90993 hsa:10891 hsa:5468 |
| hsa04662 | B cell receptor signaling pathway | 2\|118 | 76\|9228 | 0.253740433385958 | 0.816508464583768 | ENSG00000152689 ENSG00000070190 | RASGRP3 DAPP1 | hsa:25780 hsa:27071 |
| hsa04727 | GABAergic synapse | 2\|118 | 78\|9228 | 0.263246674999024 | 0.816508464583768 | ENSG00000136928 ENSG00000139112 | GABBR2 GABARAPL1 | hsa:9568 hsa:23710 |
| hsa04360 | Axon guidance | 4\|118 | 204\|9228 | 0.264308049762546 | 0.816508464583768 | ENSG00000074527 ENSG00000112902 ENSG00000196220 ENSG00000172602 | NTN4 SEMA5A SRGAP3 RND1 | hsa:59277 hsa:9037 hsa:9901 hsa:27289 |
| hsa04022 | cGMP-PKG signaling pathway | 4\|118 | 207\|9228 | 0.272815339174005 | 0.816508464583768 | ENSG00000185532 ENSG00000157613 ENSG00000116741 ENSG00000168398 | PRKG1 CREB3L1 RGS2 BDKRB2 | hsa:5592 hsa:90993 hsa:5997 hsa:624 |
| hsa03320 | PPAR signaling pathway | 2\|118 | 81\|9228 | 0.277509278565104 | 0.816508464583768 | ENSG00000134240 ENSG00000132170 | HMGCS2 PPARG | hsa:3158 hsa:5468 |
| hsa05162 | Measles | 3\|118 | 144\|9228 | 0.27982463113452 | 0.816508464583768 | ENSG00000114423 ENSG00000111335 ENSG00000137462 | CBLB OAS2 TLR2 | hsa:868 hsa:4939 hsa:7097 |
| hsa00534 | Glycosaminoglycan biosynthesis - heparan sulfate / heparin | 1\|118 | 26\|9228 | 0.284710115763288 | 0.816508464583768 | ENSG00000002587 | HS3ST1 | hsa:9957 |
| hsa05320 | Autoimmune thyroid disease | 1\|118 | 26\|9228 | 0.284710115763288 | 0.816508464583768 | ENSG00000135346 | CGA | hsa:1081 |
| hsa00592 | alpha-Linolenic acid metabolism | 1\|118 | 27\|9228 | 0.29388249202279 | 0.816508464583768 | ENSG00000188257 | PLA2G2A | hsa:5320 |
| hsa04620 | Toll-like receptor signaling pathway | 2\|118 | 85\|9228 | 0.296500250294447 | 0.816508464583768 | ENSG00000169429 ENSG00000137462 | CXCL8 TLR2 | hsa:3576 hsa:7097 |
| hsa04974 | Protein digestion and absorption | 2\|118 | 85\|9228 | 0.296500250294447 | 0.816508464583768 | ENSG00000188153 ENSG00000169031 | COL4A5 COL4A3 | hsa:1287 hsa:1285 |
| hsa04964 | Proximal tubule bicarbonate reclamation | 1\|118 | 28\|9228 | 0.302938232262037 | 0.816508464583768 | ENSG00000080493 | SLC4A4 | hsa:8671 |
| hsa05167 | Kaposi sarcoma-associated herpesvirus infection | 4\|118 | 220\|9228 | 0.310169756796471 | 0.816508464583768 | ENSG00000169429 ENSG00000159200 ENSG00000139112 ENSG00000081041 | CXCL8 RCAN1 GABARAPL1 CXCL2 | hsa:3576 hsa:1827 hsa:23710 hsa:2920 |
| hsa04115 | p53 signaling pathway | 2\|118 | 89\|9228 | 0.315420729204436 | 0.816508464583768 | ENSG00000146674 ENSG00000206075 | IGFBP3 SERPINB5 | hsa:3486 hsa:5268 |
| hsa05211 | Renal cell carcinoma | 2\|118 | 90\|9228 | 0.320134995857249 | 0.816508464583768 | ENSG00000116016 ENSG00000092969 | EPAS1 TGFB2 | hsa:2034 hsa:7042 |
| hsa00983 | Drug metabolism - other enzymes | 2\|118 | 91\|9228 | 0.324841715342928 | 0.816508464583768 | ENSG00000213366 ENSG00000241720 | GSTM2 AL158847.1 | hsa:2946 hsa:2947 |
| hsa05120 | Epithelial cell signaling in Helicobacter pylori infection | 2\|118 | 91\|9228 | 0.324841715342928 | 0.816508464583768 | ENSG00000169429 ENSG00000081041 | CXCL8 CXCL2 | hsa:3576 hsa:2920 |
| hsa00500 | Starch and sucrose metabolism | 1\|118 | 31\|9228 | 0.32942025144268 | 0.816508464583768 | ENSG00000165434 | PGM2L1 | hsa:283209 |
| hsa00591 | Linoleic acid metabolism | 1\|118 | 31\|9228 | 0.32942025144268 | 0.816508464583768 | ENSG00000188257 | PLA2G2A | hsa:5320 |
| hsa00900 | Terpenoid backbone biosynthesis | 1\|118 | 31\|9228 | 0.32942025144268 | 0.816508464583768 | ENSG00000134240 | HMGCS2 | hsa:3158 |
| hsa04713 | Circadian entrainment | 2\|118 | 94\|9228 | 0.338910922108185 | 0.816508464583768 | ENSG00000185532 ENSG00000108551 | PRKG1 RASD1 | hsa:5592 hsa:51655 |
| hsa04972 | Pancreatic secretion | 2\|118 | 94\|9228 | 0.338910922108185 | 0.816508464583768 | ENSG00000080493 ENSG00000188257 | SLC4A4 PLA2G2A | hsa:8671 hsa:5320 |
| hsa04371 | Apelin signaling pathway | 3\|118 | 161\|9228 | 0.33941954040183 | 0.816508464583768 | ENSG00000118523 ENSG00000109819 ENSG00000139112 | CTGF PPARGC1A GABARAPL1 | hsa:1490 hsa:10891 hsa:23710 |
| hsa00650 | Butanoate metabolism | 1\|118 | 33\|9228 | 0.346518226433111 | 0.816508464583768 | ENSG00000134240 | HMGCS2 | hsa:3158 |
| hsa01523 | Antifolate resistance | 1\|118 | 33\|9228 | 0.346518226433111 | 0.816508464583768 | ENSG00000125257 | ABCC4 | hsa:10257 |
| hsa00512 | Mucin type O-glycan biosynthesis | 1\|118 | 35\|9228 | 0.363183850114102 | 0.843497022062949 | ENSG00000144278 | GALNT13 | hsa:114805 |
| hsa04916 | Melanogenesis | 2\|118 | 102\|9228 | 0.375963987273993 | 0.843497022062949 | ENSG00000078401 ENSG00000157613 | EDN1 CREB3L1 | hsa:1906 hsa:90993 |
| hsa04520 | Adherens junction | 2\|118 | 103\|9228 | 0.380539589889372 | 0.843497022062949 | ENSG00000173482 ENSG00000127329 | PTPRM PTPRB | hsa:5797 hsa:5787 |
| hsa05020 | Prion diseases | 1\|118 | 38\|9228 | 0.387395269124463 | 0.843497022062949 | ENSG00000135862 | LAMC1 | hsa:3915 |
| hsa04062 | Chemokine signaling pathway | 3\|118 | 175\|9228 | 0.38837304119519 | 0.843497022062949 | ENSG00000169429 ENSG00000006606 ENSG00000081041 | CXCL8 CCL26 CXCL2 | hsa:3576 hsa:10344 hsa:2920 |
| hsa03018 | RNA degradation | 2\|118 | 105\|9228 | 0.389648768184867 | 0.843497022062949 | ENSG00000159388 ENSG00000186197 | BTG2 EDARADD | hsa:7832 hsa:2023 |
| hsa00564 | Glycerophospholipid metabolism | 2\|118 | 107\|9228 | 0.398699350938543 | 0.843497022062949 | ENSG00000188257 ENSG00000087085 | PLA2G2A ACHE | hsa:5320 hsa:43 |
| hsa04975 | Fat digestion and absorption | 1\|118 | 40\|9228 | 0.403026858828351 | 0.843497022062949 | ENSG00000188257 | PLA2G2A | hsa:5320 |
| hsa04725 | Cholinergic synapse | 2\|118 | 108\|9228 | 0.403201753721637 | 0.843497022062949 | ENSG00000157613 ENSG00000087085 | CREB3L1 ACHE | hsa:90993 hsa:43 |
| hsa05210 | Colorectal cancer | 2\|118 | 108\|9228 | 0.403201753721637 | 0.843497022062949 | ENSG00000092969 ENSG00000109321 | TGFB2 AREG | hsa:7042 hsa:374 |
| hsa04010 | MAPK signaling pathway | 5\|118 | 331\|9228 | 0.417557046573841 | 0.843497022062949 | ENSG00000152689 ENSG00000128052 ENSG00000092969 ENSG00000109321 ENSG00000120875 | RASGRP3 KDR TGFB2 AREG DUSP4 | hsa:25780 hsa:3791 hsa:7042 hsa:374 hsa:1846 |
| hsa04136 | Autophagy - other | 1\|118 | 42\|9228 | 0.418262878050111 | 0.843497022062949 | ENSG00000139112 | GABARAPL1 | hsa:23710 |
| hsa04666 | Fc gamma R-mediated phagocytosis | 2\|118 | 112\|9228 | 0.421050831451022 | 0.843497022062949 | ENSG00000078053 ENSG00000277443 | AMPH MARCKS | hsa:273 hsa:4082 |
| hsa05414 | Dilated cardiomyopathy (DCM) | 2\|118 | 112\|9228 | 0.421050831451022 | 0.843497022062949 | ENSG00000092969 ENSG00000115221 | TGFB2 ITGB6 | hsa:7042 hsa:3694 |
| hsa05206 | MicroRNAs in cancer | 3\|118 | 185\|9228 | 0.422844280771702 | 0.843497022062949 | ENSG00000092969 ENSG00000206075 ENSG00000277443 | TGFB2 SERPINB5 MARCKS | hsa:7042 hsa:5268 hsa:4082 |
| hsa04928 | Parathyroid hormone synthesis, secretion and action | 2\|118 | 113\|9228 | 0.425471375191557 | 0.843497022062949 | ENSG00000157613 ENSG00000137745 | CREB3L1 MMP13 | hsa:90993 hsa:4322 |
| hsa05216 | Thyroid cancer | 1\|118 | 44\|9228 | 0.43311325362531 | 0.843497022062949 | ENSG00000132170 | PPARG | hsa:5468 |
| hsa04931 | Insulin resistance | 2\|118 | 116\|9228 | 0.438628146777244 | 0.843497022062949 | ENSG00000157613 ENSG00000109819 | CREB3L1 PPARGC1A | hsa:90993 hsa:10891 |
| hsa04742 | Taste transduction | 1\|118 | 45\|9228 | 0.44039685946941 | 0.843497022062949 | ENSG00000136928 | GABBR2 | hsa:9568 |
| hsa04215 | Apoptosis - multiple species | 1\|118 | 46\|9228 | 0.447587665369727 | 0.843497022062949 | ENSG00000023445 | BIRC3 | hsa:330 |
| hsa04014 | Ras signaling pathway | 4\|118 | 269\|9228 | 0.452305739071936 | 0.843497022062949 | ENSG00000152689 ENSG00000128052 ENSG00000143344 ENSG00000188257 | RASGRP3 KDR RGL1 PLA2G2A | hsa:25780 hsa:3791 hsa:23179 hsa:5320 |
| hsa04979 | Cholesterol metabolism | 1\|118 | 47\|9228 | 0.454686843706295 | 0.843497022062949 | ENSG00000101670 | LIPG | hsa:9388 |
| hsa05030 | Cocaine addiction | 1\|118 | 47\|9228 | 0.454686843706295 | 0.843497022062949 | ENSG00000157613 | CREB3L1 | hsa:90993 |
| hsa05219 | Bladder cancer | 1\|118 | 48\|9228 | 0.461695552174072 | 0.843497022062949 | ENSG00000169429 | CXCL8 | hsa:3576 |
| hsa04935 | Growth hormone synthesis, secretion and action | 2\|118 | 122\|9228 | 0.464446389172565 | 0.843497022062949 | ENSG00000157613 ENSG00000146674 | CREB3L1 IGFBP3 | hsa:90993 hsa:3486 |
| hsa04740 | Olfactory transduction | 2\|118 | 123\|9228 | 0.468682483588422 | 0.843497022062949 | ENSG00000185532 ENSG00000116741 | PRKG1 RGS2 | hsa:5592 hsa:5997 |
| hsa00514 | Other types of O-glycan biosynthesis | 1\|118 | 50\|9228 | 0.475446117949621 | 0.843497022062949 | ENSG00000144278 | GALNT13 | hsa:114805 |
| hsa00565 | Ether lipid metabolism | 1\|118 | 51\|9228 | 0.48219021885199 | 0.843497022062949 | ENSG00000188257 | PLA2G2A | hsa:5320 |
| hsa04913 | Ovarian steroidogenesis | 1\|118 | 51\|9228 | 0.48219021885199 | 0.843497022062949 | ENSG00000135346 | CGA | hsa:1081 |
| hsa00600 | Sphingolipid metabolism | 1\|118 | 52\|9228 | 0.488848337428373 | 0.843497022062949 | ENSG00000177076 | ACER2 | hsa:340485 |
| hsa00970 | Aminoacyl-tRNA biosynthesis | 1\|118 | 52\|9228 | 0.488848337428373 | 0.843497022062949 | novel.282 | - | hsa:54938 |
| hsa05225 | Hepatocellular carcinoma | 3\|118 | 205\|9228 | 0.489639734758492 | 0.843497022062949 | ENSG00000213366 ENSG00000092969 ENSG00000241720 | GSTM2 TGFB2 AL158847.1 | hsa:2946 hsa:7042 hsa:2947 |
| hsa00140 | Steroid hormone biosynthesis | 1\|118 | 54\|9228 | 0.501910961821267 | 0.85414023094664 | ENSG00000101846 | STS | hsa:412 |
| hsa04071 | Sphingolipid signaling pathway | 2\|118 | 132\|9228 | 0.505899940371956 | 0.85414023094664 | ENSG00000177076 ENSG00000168398 | ACER2 BDKRB2 | hsa:340485 hsa:624 |
| hsa00250 | Alanine, aspartate and glutamate metabolism | 1\|118 | 55\|9228 | 0.508317600856049 | 0.85414023094664 | ENSG00000086205 | FOLH1 | hsa:2346 |
| hsa04730 | Long-term depression | 1\|118 | 56\|9228 | 0.514642524337897 | 0.857364293124299 | ENSG00000185532 | PRKG1 | hsa:5592 |
| hsa00330 | Arginine and proline metabolism | 1\|118 | 58\|9228 | 0.527051345571084 | 0.857364293124299 | ENSG00000130066 | SAT1 | hsa:6303 |
| hsa00280 | Valine, leucine and isoleucine degradation | 1\|118 | 59\|9228 | 0.533137271549558 | 0.857364293124299 | ENSG00000134240 | HMGCS2 | hsa:3158 |
| hsa04622 | RIG-I-like receptor signaling pathway | 1\|118 | 61\|9228 | 0.545077129764818 | 0.857364293124299 | ENSG00000169429 | CXCL8 | hsa:3576 |
| hsa04217 | Necroptosis | 3\|118 | 223\|9228 | 0.546454304113595 | 0.857364293124299 | ENSG00000162909 ENSG00000138378 ENSG00000023445 | CAPN2 STAT4 BIRC3 | hsa:824 hsa:6775 hsa:330 |
| hsa04120 | Ubiquitin mediated proteolysis | 3\|118 | 224\|9228 | 0.54950417554295 | 0.857364293124299 | ENSG00000114423 ENSG00000101871 ENSG00000023445 | CBLB MID1 BIRC3 | hsa:868 hsa:4281 hsa:330 |
| hsa04670 | Leukocyte transendothelial migration | 2\|118 | 147\|9228 | 0.564093626580978 | 0.857364293124299 | ENSG00000179776 ENSG00000116701 | CDH5 NCF2 | hsa:1003 hsa:4688 |
| hsa05169 | Epstein-Barr virus infection | 3\|118 | 230\|9228 | 0.56755231348809 | 0.857364293124299 | ENSG00000168032 ENSG00000111335 ENSG00000137462 | ENTPD3 OAS2 TLR2 | hsa:956 hsa:4939 hsa:7097 |
| hsa04919 | Thyroid hormone signaling pathway | 2\|118 | 148\|9228 | 0.567796372389162 | 0.857364293124299 | ENSG00000159200 ENSG00000211448 | RCAN1 DIO2 | hsa:1827 hsa:1734 |
| hsa04927 | Cortisol synthesis and secretion | 1\|118 | 65\|9228 | 0.568055955623967 | 0.857364293124299 | ENSG00000157613 | CREB3L1 | hsa:90993 |
| hsa05031 | Amphetamine addiction | 1\|118 | 65\|9228 | 0.568055955623967 | 0.857364293124299 | ENSG00000157613 | CREB3L1 | hsa:90993 |
| hsa00561 | Glycerolipid metabolism | 1\|118 | 67\|9228 | 0.579109967220659 | 0.857364293124299 | ENSG00000101670 | LIPG | hsa:9388 |
| hsa04066 | HIF-1 signaling pathway | 2\|118 | 152\|9228 | 0.582382647187447 | 0.857364293124299 | ENSG00000078401 ENSG00000186197 | EDN1 EDARADD | hsa:1906 hsa:2023 |
| hsa00240 | Pyrimidine metabolism | 1\|118 | 68\|9228 | 0.584531321206901 | 0.857364293124299 | ENSG00000168032 | ENTPD3 | hsa:956 |
| hsa04370 | VEGF signaling pathway | 1\|118 | 69\|9228 | 0.589883428641135 | 0.857364293124299 | ENSG00000128052 | KDR | hsa:3791 |
| hsa04924 | Renin secretion | 1\|118 | 69\|9228 | 0.589883428641135 | 0.857364293124299 | ENSG00000078401 | EDN1 | hsa:1906 |
| hsa05133 | Pertussis | 1\|118 | 69\|9228 | 0.589883428641135 | 0.857364293124299 | ENSG00000169429 | CXCL8 | hsa:3576 |
| hsa05205 | Proteoglycans in cancer | 3\|118 | 238\|9228 | 0.590927364608034 | 0.857364293124299 | ENSG00000128052 ENSG00000092969 ENSG00000137462 | KDR TGFB2 TLR2 | hsa:3791 hsa:7042 hsa:7097 |
| hsa05032 | Morphine addiction | 1\|118 | 70\|9228 | 0.595167166540507 | 0.857364293124299 | ENSG00000136928 | GABBR2 | hsa:9568 |
| hsa04015 | Rap1 signaling pathway | 3\|118 | 240\|9228 | 0.59664484368501 | 0.857364293124299 | ENSG00000152689 ENSG00000128052 ENSG00000082074 | RASGRP3 KDR FYB1 | hsa:25780 hsa:3791 hsa:2533 |
| hsa04920 | Adipocytokine signaling pathway | 1\|118 | 71\|9228 | 0.600383400909171 | 0.857364293124299 | ENSG00000109819 | PPARGC1A | hsa:10891 |
| hsa05135 | Yersinia infection | 2\|118 | 164\|9228 | 0.623974419669415 | 0.857364293124299 | ENSG00000169429 ENSG00000082074 | CXCL8 FYB1 | hsa:3576 hsa:2533 |
| hsa04068 | FoxO signaling pathway | 2\|118 | 165\|9228 | 0.627293655586325 | 0.857364293124299 | ENSG00000092969 ENSG00000139112 | TGFB2 GABARAPL1 | hsa:7042 hsa:23710 |
| hsa04072 | Phospholipase D signaling pathway | 2\|118 | 165\|9228 | 0.627293655586325 | 0.857364293124299 | ENSG00000197959 ENSG00000169429 | DNM3 CXCL8 | hsa:26052 hsa:3576 |
| hsa04658 | Th1 and Th2 cell differentiation | 1\|118 | 77\|9228 | 0.630310375769922 | 0.857364293124299 | ENSG00000138378 | STAT4 | hsa:6775 |
| hsa04917 | Prolactin signaling pathway | 1\|118 | 77\|9228 | 0.630310375769922 | 0.857364293124299 | ENSG00000135346 | CGA | hsa:1081 |
| hsa04934 | Cushing syndrome | 2\|118 | 166\|9228 | 0.630590387822808 | 0.857364293124299 | ENSG00000157613 ENSG00000108551 | CREB3L1 RASD1 | hsa:90993 hsa:51655 |
| hsa04911 | Insulin secretion | 1\|118 | 78\|9228 | 0.635077436818895 | 0.857364293124299 | ENSG00000157613 | CREB3L1 | hsa:90993 |
| hsa04970 | Salivary secretion | 1\|118 | 78\|9228 | 0.635077436818895 | 0.857364293124299 | ENSG00000185532 | PRKG1 | hsa:5592 |
| hsa04144 | Endocytosis | 4\|118 | 343\|9228 | 0.644253370522416 | 0.857364293124299 | ENSG00000078053 ENSG00000197959 ENSG00000153071 ENSG00000114423 | AMPH DNM3 DAB2 CBLB | hsa:273 hsa:26052 hsa:1601 hsa:868 |
| hsa04910 | Insulin signaling pathway | 2\|118 | 172\|9228 | 0.649899952836992 | 0.857364293124299 | ENSG00000109819 ENSG00000114423 | PPARGC1A CBLB | hsa:10891 hsa:868 |
| hsa05160 | Hepatitis C | 2\|118 | 173\|9228 | 0.653040092563123 | 0.857364293124299 | ENSG00000111335 ENSG00000134321 | OAS2 RSAD2 | hsa:4939 hsa:91543 |
| hsa00310 | Lysine degradation | 1\|118 | 85\|9228 | 0.666782706714823 | 0.857364293124299 | ENSG00000008311 | AASS | hsa:10157 |
| hsa05152 | Tuberculosis | 2\|118 | 178\|9228 | 0.668408743924721 | 0.857364293124299 | ENSG00000092969 ENSG00000137462 | TGFB2 TLR2 | hsa:7042 hsa:7097 |
| hsa04137 | Mitophagy - animal | 2\|118 | 180\|9228 | 0.674402262563865 | 0.857364293124299 | ENSG00000139112 ENSG00000123240 | GABARAPL1 OPTN | hsa:23710 hsa:10133 |
| hsa04915 | Estrogen signaling pathway | 2\|118 | 180\|9228 | 0.674402262563865 | 0.857364293124299 | ENSG00000157613 ENSG00000136928 | CREB3L1 GABBR2 | hsa:90993 hsa:9568 |
| hsa04921 | Oxytocin signaling pathway | 2\|118 | 180\|9228 | 0.674402262563865 | 0.857364293124299 | ENSG00000159200 ENSG00000116741 | RCAN1 RGS2 | hsa:1827 hsa:5997 |
| hsa04721 | Synaptic vesicle cycle | 1\|118 | 87\|9228 | 0.675328705349072 | 0.857364293124299 | ENSG00000197959 | DNM3 | hsa:26052 |
| hsa04140 | Autophagy - animal | 3\|118 | 270\|9228 | 0.676103324577088 | 0.857364293124299 | ENSG00000139112 ENSG00000078804 ENSG00000062716 | GABARAPL1 TP53INP2 VMP1 | hsa:23710 hsa:58476 hsa:81671 |
| hsa04971 | Gastric acid secretion | 1\|118 | 88\|9228 | 0.679519845571018 | 0.857364293124299 | ENSG00000147606 | SLC26A7 | hsa:115111 |
| hsa04714 | Thermogenesis | 4\|118 | 360\|9228 | 0.68170916965493 | 0.857364293124299 | ENSG00000185532 ENSG00000157613 ENSG00000109819 ENSG00000132170 | PRKG1 CREB3L1 PPARGC1A PPARG | hsa:5592 hsa:90993 hsa:10891 hsa:5468 |
| hsa04210 | Apoptosis | 2\|118 | 185\|9228 | 0.689005489772178 | 0.861256862215223 | ENSG00000162909 ENSG00000023445 | CAPN2 BIRC3 | hsa:824 hsa:330 |
| hsa04145 | Phagosome | 2\|118 | 192\|9228 | 0.708550398798517 | 0.866534959535384 | ENSG00000116701 ENSG00000137462 | NCF2 TLR2 | hsa:4688 hsa:7097 |
| hsa04750 | Inflammatory mediator regulation of TRP channels | 1\|118 | 96\|9228 | 0.711173693319632 | 0.866534959535384 | ENSG00000168398 | BDKRB2 | hsa:624 |
| hsa05212 | Pancreatic cancer | 1\|118 | 96\|9228 | 0.711173693319632 | 0.866534959535384 | ENSG00000092969 | TGFB2 | hsa:7042 |
| hsa01521 | EGFR tyrosine kinase inhibitor resistance | 1\|118 | 97\|9228 | 0.714905789704683 | 0.866534959535384 | ENSG00000128052 | KDR | hsa:3791 |
| hsa04660 | T cell receptor signaling pathway | 1\|118 | 98\|9228 | 0.718590064900702 | 0.866534959535384 | ENSG00000114423 | CBLB | hsa:868 |
| hsa05235 | PD-L1 expression and PD-1 checkpoint pathway in cancer | 1\|118 | 99\|9228 | 0.722227126493441 | 0.866534959535384 | ENSG00000137462 | TLR2 | hsa:7097 |
| hsa05220 | Chronic myeloid leukemia | 1\|118 | 100\|9228 | 0.725817574414766 | 0.866534959535384 | ENSG00000092969 | TGFB2 | hsa:7042 |
| hsa04350 | TGF-beta signaling pathway | 1\|118 | 103\|9228 | 0.736315122659648 | 0.866534959535384 | ENSG00000092969 | TGFB2 | hsa:7042 |
| hsa04925 | Aldosterone synthesis and secretion | 1\|118 | 103\|9228 | 0.736315122659648 | 0.866534959535384 | ENSG00000157613 | CREB3L1 | hsa:90993 |
| hsa04540 | Gap junction | 1\|118 | 104\|9228 | 0.739724965457035 | 0.866534959535384 | ENSG00000185532 | PRKG1 | hsa:5592 |
| hsa04912 | GnRH signaling pathway | 1\|118 | 104\|9228 | 0.739724965457035 | 0.866534959535384 | ENSG00000135346 | CGA | hsa:1081 |
| hsa04724 | Glutamatergic synapse | 1\|118 | 106\|9228 | 0.746414030479029 | 0.868290075237986 | ENSG00000164418 | GRIK2 | hsa:2898 |
| hsa05412 | Arrhythmogenic right ventricular cardiomyopathy (ARVC) | 1\|118 | 107\|9228 | 0.749694357644505 | 0.868290075237986 | ENSG00000115221 | ITGB6 | hsa:3694 |
| hsa04625 | C-type lectin receptor signaling pathway | 1\|118 | 111\|9228 | 0.762400199430069 | 0.876661792665304 | ENSG00000114423 | CBLB | hsa:868 |
| hsa05100 | Bacterial invasion of epithelial cells | 1\|118 | 112\|9228 | 0.765475418961411 | 0.876661792665304 | ENSG00000197959 | DNM3 | hsa:26052 |
| hsa05215 | Prostate cancer | 1\|118 | 115\|9228 | 0.774466265554866 | 0.87957951508201 | ENSG00000157613 | CREB3L1 | hsa:90993 |
| hsa05132 | Salmonella infection | 3\|118 | 317\|9228 | 0.776604352340702 | 0.87957951508201 | ENSG00000169429 ENSG00000023445 ENSG00000137462 | CXCL8 BIRC3 TLR2 | hsa:3576 hsa:330 hsa:7097 |
| hsa01230 | Biosynthesis of amino acids | 1\|118 | 118\|9228 | 0.783115244358787 | 0.882080357656876 | ENSG00000186197 | EDARADD | hsa:2023 |
| hsa04728 | Dopaminergic synapse | 1\|118 | 133\|9228 | 0.821666846736362 | 0.915265658152647 | ENSG00000157613 | CREB3L1 | hsa:90993 |
| hsa05163 | Human cytomegalovirus infection | 2\|118 | 251\|9228 | 0.835787702102081 | 0.915265658152647 | ENSG00000157613 ENSG00000169429 | CREB3L1 CXCL8 | hsa:90993 hsa:3576 |
| hsa04611 | Platelet activation | 1\|118 | 141\|9228 | 0.839364074976982 | 0.915265658152647 | ENSG00000185532 | PRKG1 | hsa:5592 |
| hsa04810 | Regulation of actin cytoskeleton | 2\|118 | 257\|9228 | 0.845452133831949 | 0.915265658152647 | ENSG00000168398 ENSG00000115221 | BDKRB2 ITGB6 | hsa:624 hsa:3694 |
| hsa04550 | Signaling pathways regulating pluripotency of stem cells | 1\|118 | 144\|9228 | 0.845541689647236 | 0.915265658152647 | ENSG00000105991 | HOXA1 | hsa:3198 |
| hsa04722 | Neurotrophin signaling pathway | 1\|118 | 145\|9228 | 0.847548083374848 | 0.915265658152647 | ENSG00000111348 | ARHGDIB | hsa:397 |
| hsa05166 | Human T-cell leukemia virus 1 infection | 2\|118 | 261\|9228 | 0.851607937629675 | 0.915265658152647 | ENSG00000157613 ENSG00000092969 | CREB3L1 TGFB2 | hsa:90993 hsa:7042 |
| hsa04630 | JAK-STAT signaling pathway | 1\|118 | 148\|9228 | 0.853413512155146 | 0.915265658152647 | ENSG00000138378 | STAT4 | hsa:6775 |
| hsa05226 | Gastric cancer | 1\|118 | 148\|9228 | 0.853413512155146 | 0.915265658152647 | ENSG00000092969 | TGFB2 | hsa:7042 |
| hsa00230 | Purine metabolism | 1\|118 | 153\|9228 | 0.862696029863834 | 0.915265658152647 | ENSG00000168032 | ENTPD3 | hsa:956 |
| hsa05131 | Shigellosis | 3\|118 | 375\|9228 | 0.864141032719657 | 0.915265658152647 | ENSG00000169429 ENSG00000162909 ENSG00000139112 | CXCL8 CAPN2 GABARAPL1 | hsa:3576 hsa:824 hsa:23710 |
| hsa04261 | Adrenergic signaling in cardiomyocytes | 1\|118 | 155\|9228 | 0.866243669570561 | 0.915265658152647 | ENSG00000157613 | CREB3L1 | hsa:90993 |
| hsa05130 | Pathogenic Escherichia coli infection | 2\|118 | 276\|9228 | 0.872768277516088 | 0.915265658152647 | ENSG00000169429 ENSG00000006453 | CXCL8 BAIAP2L1 | hsa:3576 hsa:55971 |
| hsa04142 | Lysosome | 1\|118 | 162\|9228 | 0.877959536395621 | 0.915265658152647 | ENSG00000119899 | SLC17A5 | hsa:26503 |
| hsa04110 | Cell cycle | 1\|118 | 163\|9228 | 0.879547973932056 | 0.915265658152647 | ENSG00000092969 | TGFB2 | hsa:7042 |
| hsa04310 | Wnt signaling pathway | 1\|118 | 169\|9228 | 0.888657132672386 | 0.92007430402949 | ENSG00000133067 | LGR6 | hsa:59352 |
| hsa01200 | Carbon metabolism | 1\|118 | 183\|9228 | 0.907341244977866 | 0.93469826743951 | ENSG00000186197 | EDARADD | hsa:2023 |
| hsa04020 | Calcium signaling pathway | 1\|118 | 202\|9228 | 0.927819466146617 | 0.94892138149 | ENSG00000168398 | BDKRB2 | hsa:624 |
| hsa05034 | Alcoholism | 1\|118 | 205\|9228 | 0.930613846126931 | 0.94892138149 | ENSG00000157613 | CREB3L1 | hsa:90993 |
| hsa04141 | Protein processing in endoplasmic reticulum | 1\|118 | 210\|9228 | 0.935034727126732 | 0.94892138149 | ENSG00000162909 | CAPN2 | hsa:824 |
| hsa04932 | Non-alcoholic fatty liver disease (NAFLD) | 1\|118 | 225\|9228 | 0.946689741754385 | 0.956016734283985 | ENSG00000169429 | CXCL8 | hsa:3576 |
| hsa05170 | Human immunodeficiency virus 1 infection | 1\|118 | 242\|9228 | 0.957409116961351 | 0.96210229890724 | ENSG00000137462 | TLR2 | hsa:7097 |
| hsa05203 | Viral carcinogenesis | 1\|118 | 275\|9228 | 0.972487365374527 | 0.972487365374527 | ENSG00000157613 | CREB3L1 | hsa:90993 |
